# Editorial: Women in molecular mechanisms of aging

**DOI:** 10.3389/fragi.2024.1471233

**Published:** 2024-08-05

**Authors:** Aurélie Ledreux, Consuelo Borrás

**Affiliations:** ^1^ Department of Neurosurgery, School of Medicine, University of Colorado Anschutz Medical Campus, Aurora, CO, United States; ^2^ MiniAging Research Group, Department of Physiology, Faculty of Medicine, University of Valencia, Valencia, Spain

**Keywords:** aging, lifespan, *C. elegans*, Klotho, vascular aging, mTOR, nanohistology

This *Women in molecular mechanisms of aging* Research Topic highlights research studies and methods focused on aging. This Research Topic includes 7 articles, spanning various areas of aging research but with a common goal of deepening the current understanding of mechanisms and pathways involved in aging. From *in vitro* studies (Hartmann et al.; Wang et al.; Jiang and Ji; Huang et al.), to a novel *C. elegans* Observatory (Kerr et al.), or a clinical study (Collins et al.) and a research method study proposing a novel way to estimate lifespan (Adelöf et al.).

Lifespan analyses are important in determining the effect of a gene or the efficacy of a drug intervention and other therapeutic approaches to elucidate the underlying molecular mechanisms of aging. Adelöf et al. propose a novel method to better estimate lifespan in aging studies. The aging process strongly intertwines with disease onset and progression, which can complicate the rigorous interpretation of aging mechanisms since diseased animals are often excluded in traditional methods to estimate lifespan. The “survival-span method” proposed by this team of women scientists integrates the effect of decreased health by including euthanized “diseased” animals together with animals that died from “natural causes.” This is significant for aging studies since the exclusion of diseased animals from a lifespan analysis can decrease the power of the study and might provide misleading data on actual genes or pathways involved in aging (Adelöf et al.).

While various cellular systems have been implicated in regulating aging, it remains unclear how these processes cause the aging process to unfold. *C. elegans* short lifespan and ease of genetic manipulation has made it a premier model organism for aging studies, leading to the identification of hundreds of genes that modulate lifespan. Kerr et al. from Calico Life Science have created an automated system for monitoring the behavior of group-housed *C. elegans* throughout their lifespans. The *C. elegans* Observatory makes it possible to study known and to-be-discovered lifespan genes that influence how animals age in a high-throughput yet approachable manner, opening the avenue for a deeper exploration of aging trajectories.

*In vitro* studies have yielded a great deal of knowledge on aging processes. Yet, comparison between studies is rendered challenging not only by the use of different cell lines but is also hampered by the lack of a uniform panel of age markers. Hartmann et al. from Rostock University Medical Center in Germany highlight the heterogeneity of single markers and the flaws of using individual marker expression. Instead, they designed a panel of markers that are sensitive to various aging conditions and can be used to assess different aspects of aging. They propose an “AgeScore” including primary aging markers as well as antagonist aging markers that can be used in any *in vitro* cell culture system to estimate the biological age of cells in culture.

The skin is a complex and multi-layered connective tissue and illustrates the naturally occurring changes associated with age, resulting from both chronological and environmental factors. Huang et al. used Atomic-Force Microscopy (AFM) quantitative nanohistology to gain a better understanding of the phenotypic diversity of collagen across age at the nanoscale level. Their study provides new insights into the structural and functional properties of the skin and could drive a new field of quantitative nanohistology.

The mammalian target of rapamycin (mTOR) is a critical protein kinase that regulates cell growth and metabolism, and overactive mTOR has been implicated in cancer, aging, and neurodegeneration. Wang et al. investigated the mechanisms by which simufilam, a small oral molecule drug candidate for Alzheimer’s disease (AD) can reduce mTOR activity in the lymphocytes of people with AD who participated in a phase 2 clinical trial of simulifam.

Klotho is another protein that has been associated with healthy aging, shown to promote longevity and provide cardiovascular and neuroprotective effects. Obesity and overweight can accelerate aging mechanisms through, e.g., increased oxidative stress and inflammation or DNA damage. Blood levels of Klotho are lower in obese individuals, highlighting the accelerated aging process at play. Collins et al. from Duke Molecular Physiology Institute found that, in middle-aged obese people who underwent weight loss treatment (diet) associated or not with physical activity, Klotho levels increased significantly in participants with weight loss ≥10% compared to 5% across 12 months. These findings suggest that weight loss is favorably associated with changes in Klotho concentrations, potentially counteracting the accelerated aging effects of obesity.

Vascular aging is considered a prominent factor in the global prevalence of cardiovascular diseases. Jiang and Ji from the Department of Biomedical Engineering at Purdue Indiana University investigated the role of progerin in wound healing and proliferation of endothelial cells (EC) submitted to shear stress. Progerin, a mutant truncated form of lamin A, is involved in Hutchinson-Gilford progeria syndrome, but it has also been detected in healthy donor cells. Progerin activates a proinflammatory response in vascular cells and accelerates senescence, suggesting a role for progerin in vascular aging. Steady laminar flow, by regulating EC proliferation and remodeling processes, can protect against atherosclerosis. The study by Jiang and Ji showed that progerin-expressing ECs exposed to physiological levels of laminar shear stress exhibit delayed wound healing, which might affect atherosclerosis in older individuals.

